# Osteoclastic giant cell tumor of the pancreas with synchronous jejunal gastrointestinal stromal tumor: A case report

**DOI:** 10.1016/j.amsu.2019.11.008

**Published:** 2019-11-23

**Authors:** Waad Farhat, Houssem Ammar, Abdelkader Mizouni, Fathia harrabi, Amal Bouazzi, Eya hammami, Rahul Gupta, Mohamed amine said, Mohamed Ben Mabrouk, Ali Ben Ali

**Affiliations:** a Department of Gastrointestinal Surgery, University of Sousse, Sahloul Hospital, Sousse, Tunisia; b Department of Gastroenterology, University of Sousse, Sahloul Hospital, Sousse, Tunisia; c Department of Gastrointestinal Surgery, Synergy Institute of Medical Sciences, Dehradun, India

**Keywords:** OGCT of the pancreas, Jejunal GIST, OGCT, Osteoclastic giant cell tumor, GIST, Stromal gastrointestinal tumor, CT, computed tomography, FNA, fine needle aspiration, EMA, epithelial membrane antigen

## Abstract

Osteoclastic giant cell tumor of the pancreas is a rare aggressive tumor, counting for 2–7% of all pancreatic cancers. Surgery is considered the most appropriate treatment. We report a case of a 84-year-old man with incidentally detected 11cm tumor in the pancreatic tail and another 6 cm tumor located in the jejunum on abdominal computed tomography. The patient underwent distal pancreatectomy with splenectomy along with segmental resection of the tumor bearing part of the jejunum. On histological examination, osteoclast-like giant cells with some areas of metaplastic bone were observed which confirmed the diagnosis of osteoclastic tumor of the pancreas. The jejunal tumor was strongly c-kit positive on immunohistochemistry which confirmed the diagnosis of GIST. On the last follow up at 2 years after surgery, there is no evidence of recurrence or distant metastasis. Pancreatic OGCT has a better prognosis after resection than pancreatic adenocarcinoma. Its co-existence jejunal GIST, as seen in the index case, has not been reported in the English literature till date.

## Highlights

• Osteoclastic giant cell tumor of the pancreas is a rare aggressive tumor, counting 2–7% of all pancreatic cancers.
• Surgery is considered the most appropriate treatment.
• Pancreatic OGCT has a better prognosis, after resection than pancreatic adenocarcinoma.
• Its co-existence jejunal GIST, as seen in the index case, has not been reported in the English literature till date.

## 1 Introduction

Osteoclastic giant cell tumor (OGCT) of the pancreas is a rare aggressive tumor, which accounts for 2–7% of all pancreatic cancers [1]. In 2010, the World Health Organization classified these tumors as variants of pancreatic ductal adenocarcinoma under the heading “undifferentiated carcinoma with osteoclastic giant cells,” and they have been identified as fundamentally epithelial-derived tumors with mesenchymal differentiation [1,2]. Here, we report a case of OGCT of the pancreas associated with jejunal gastrointestinal stromal tumor (GIST). This is the first case report of the co-existence of these two tumors in the English literature till date. This case has been reported in line with the SCARE criteria [3].

## 2 Case report

**Fig. 1:**
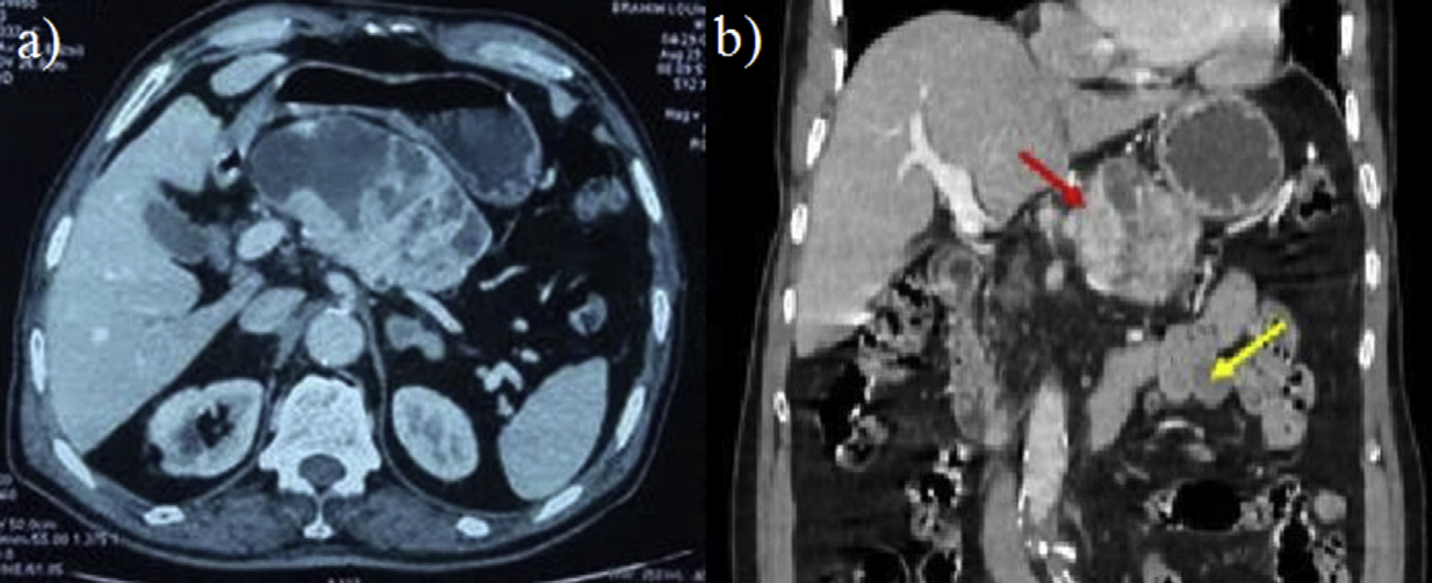
CT abdomen: (a) axial section showing the pancreatic tumor, (b) coronal section showing the pancreatic tumor (red arrow) and the jejunal GIST (yellow arrow). (For interpretation of the references to colour in this figure legend, the reader is referred to the Web version of this article.)

A 84 -years –old lady with presented with epigastric pain and vomiting for 3 months. The pain was dull aching in nature, paroxysmal, unrelated to food and without radiation. There was no history of associated hematemesis, or weight loss. She had past history of myocardial infarction 3 years back for which she was taking oral antiplatelet therapy, Physical examination revealed an epigastric intra-abdominal mass measuring around 4 × 4 cm, firm in consistency with rounded borders. Routine blood investigations and tumor markers (serum CA 19-9, CEA) were within normal range. Upper gastrointestinal endoscopy was unremarkable. Abdominal ultrasound (US) showed a 10 × 7 cm well-defined, round-like, mixed cystic and solid mass in the body of the pancreas. On contrast enhanced computed tomography of the abdomen (CT), a well-defined solid-cystic mass of 11 × 7 × 6 cm was found involving the body and tail of the pancreas (Fig. 1). The cystic lesion had multiple septae which showed contrast enhancement with solid areas without involvement of the surrounding structures such as bile duct, splenic vessels, stomach and transverse colon. The main pancreatic duct could not be separately identified from the tumor. Additionally, there was a 6 cm tumor in close relation with the proximal jejunum (Fig. 1). Based on the imaging findings, a provisional diagnosis of malignant/borderline malignant pancreatic tumor such as solid pseudopapillary tumor or neuroendocrine tumor or GIST along with benign jejunal tumor was made. As the lesions were resectable on CT and patient was symptomatic, further investigations like endoscopic ultrasound (EUS) or magnetic resonance imaging (MRI) were not performed and the patient was planned for surgical resection. On laparotomy, the pancreatic tumor was found to be involving the body and tail of the pancreas for which distal pancreatectomy with splenectomy was performed. Another tumor was arising from the proximal jejunum for which segmental resection with end to end anastomosis was performed. The operative time was 90 minutes and estimated blood loss was 50 ml. Postoperative course of the patient was uneventful. The hospital stay was 4 days.

**Fig. 2:**
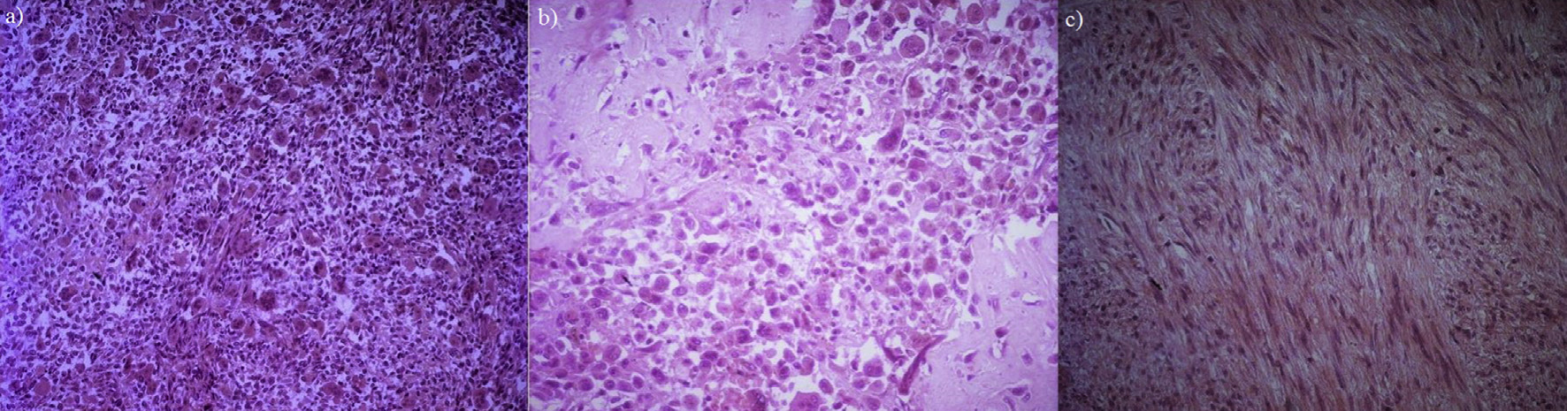
Microscopic examination: (a) pancreatic tumor showing a predominant giant osteoclastic like cells component (HEx100), (b) foci of osteoid bone was present within the pancreatic tumor (HEx400), (c) jejunal gastrointestinal stromal tumor made of spindle cells with ovoid shaped nuclei arranged in fascicles (HEx200).

Macroscopically, it was 11-cm well-defined tumor involving the body and tail of the pancreas. On cut section, areas of necrosis could be observed. On histological examination, the tumor cells were fusiform, epithelioid or polygonal in appearance with atypical nuclei. Osteoclast-like giant cells were observed within the stroma, consisting the major component of the tumor. (Fig. 2). Some areas of metaplastic bone were also noticed. Eight lymph nodes were examined and all were tumor-free. All the resection margins were free of tumor. The jejunal tumor showed spindle cells with ovoid shaped nuclei and were arranged in fasciculate pattern suggestive of gastrointestinal stromal tumor (GIST) (Fig. 2). On immunochemistry, intestinal GIST was positive for cKIT and DOG1 while the pancreatic OGCT was negative for these markers and strongly expressed CD68, a specific marker of the macrophage/monocyte/histiocyte phagocytic activities suggesting no similarities between these two tumors on histology and immunohistochemistry.

On the last follow up at 2 years after surgery, there is no evidence of recurrence or distant metastasis on CT abdomen and pelvis.

## 3 Discussion

Osteoclastic giant cell tumor (OGCT) is a rare, distinctive, malignant pancreatic tumor that is typically large, frequently has a pancreatic ductal adenocarcinoma component, and has 3 classical cell types: osteoclastic, pleomorphic, and mixed. Pancreatic OGCT is more frequent in women with mean age of 58 years [2]. The symptoms are mostly nonspecific. Characteristically, OGCs are large at presentation and show focal hemorrhage and necrosis, but seem slow to give rise to metastases [[1], [2], [3], [4]]. Radiologically, they appear as solid-cystic tumors with delayed enhancement and difficult to differentiate from other pancreatic tumors. Reid et al. have reported a series of 15 patients with OGCT, diagnosed via endoscopic ultrasound or CT guided fine needle aspiration (FNA), demonstrating the efficacy of the technique in this setting [2]. Lesions that appear to be surgically resectable should undergo operative intervention. Histological examination reveals numerous osteoclast-like giant cells set in a sarcomatous stroma, the appearances being similar to those seen in giant cell tumors of bone. Fusiform and pleomorphic cells express the cytokeratin, the epithelial membrane antigen (EMA) and the vimentin antigens. The osteoclast-like giant cells express the CD68 and rarely the vimentin [[2], [3], [4]]. CD68 is highly expressed in macrophages, monocytes and histiocytes. It is also called as phagocyte marker. But the exact origin of these osteoclastic cells in pancreatic tumor is not known. They are distinct from pleomorphic giant cell carcinomas of the pancreas and have a slightly better prognosis after resection than ductal adenocarcinoma [[2], [3], [4], [5]].

GIST are the most common mesenchymal tumors of the gastrointestinal tract and usually show cKIT and DOG1 positivity on immunohistochemistry. The existence of pancreatic OGCT and jejunal GIST, as seen in the index case, has not been reported in the English literature till date. However, there are some reported cases of the co-existence of GIST having osteoclastic –like giant cells associated with other abdominal tumors [6,7]. Therefore, these findings lead us to consider the hypothesis of common etiology, making the synchronous occurrence of GIST and other abdominal malignancy not just a coincidence. The prognosis in these patients is predominantly determined by the other malignancy and not influenced by GIST [5].

## 4 Conclusion

Pancreatic OGCT are rare tumors, their association with jejunal GIST is even rarer. This is the first case to be reported in literature associating these two tumors. Its prognosis is better than pancreatic adenocarcinoma and its evolution is generally favorable after total surgical resection.

### Provenance and peer review

Not commissioned, externally peer reviewed.

## Sources of funding

This study has not received any funding.

## Ethical approval

The study was approved by Ethics Committee.

## Author contribution

Study concept or design – MBM, HA.

Data collection – HA, WF, RG.

Data interpretation – MBM, AB, EH.

Literature review – WF, SL, EH,ABA.

Drafting of the paper – HA, MBM, SL.

Editing of the paper – MBM, WF,AM.

## Registration of research studies

1. Name of the registry:

2. Unique Identifying number or registration ID:

3. Hyperlink to the registration (must be publicly accessible):

As this was a case report and not a clinical trial, this study does not require registration.

## Guarantor

Waad farhat.

## Financial support

None.

## Informed consent

The patient provided informed written consent prior to submission of this manuscript.

## Declaration of competing interestCOI

The authors declare no conflict of interest.

## Patient consent

Informed consent was taken from the patients for this study.

## Ethical approval

All procedures performed in studies involving human participants were in accordance with the ethical standards of the institutional research committee.

## Declaration of competing interest

The authors declare that they have no conflict of interest.
